# The role of urothelial PIEZO ion channels in urinary function

**DOI:** 10.64898/2026.10.10.758063

**Published:** 2026-10-16

**Authors:** Yasmeen M. F. Hamed, Max A. Odem, Shriya Pendyala, Tammy B. Kwok, Kara L. Marshall

## Abstract

The urothelium is increasingly recognized as a dynamic sensory interface that contributes to the regulation of urinary tract function. Mechanosensitive PIEZO ion channels expressed by urothelial cells have been proposed to play important roles in bladder fullness sensation and urinary reflexes. However, the functional contribution of urothelial PIEZO channels to these processes remains unclear. Here, we investigated the consequences of urothelial *Piezo1* and *Piezo2* deletion using the commonly used *Upk2-cre* transgenic line. Surprisingly, combined deletion of urothelial PIEZO channels did not alter urinary behavior, and mice lacking urothelial PIEZO1/2 exhibited normal bladder pressure-volume relationships, indicating preserved bladder filling and voiding function. Notably, *Upk2-cre* ; *Piezo1* ^*fl/fl*^ mice displayed enhanced external urethral sphincter reflex activity during voiding, suggesting that loss of PIEZO1 from cells targeted by *Upk2-cre* may influence sensory signaling outside of the bladder urothelium. Consistent with this possibility, we identified substantial off-target *Cre* expression in dorsal root ganglia. These findings demonstrate that the *Upk2-cre* line is not restricted to the urothelium and therefore raises important considerations for interpreting associated phenotypes attributed specifically to urothelial gene deletion. Biologically, this raises the possibility that PIEZO1 does not influence bladder pressure-volume handling but might contribute to urinary reflexes.

**HIGHLIGHTS:**

- Urothelial *Piezo1/2* deletion using the *Upk2-cre* line does not alter urinary behavior
- Mice lacking urothelial PIEZO channels show normal bladder pressure-volume handling
- Voiding urethral reflexes are enhanced in *Upk2-cre* ^*+*^ ; *Piezo1* ^*fl/fl*^ mice
- *Upk2-cre* line shows off-target *cre* expression in dorsal root ganglia

